# Are growing pains a parasomnia

**DOI:** 10.1186/1546-0096-10-S1-A78

**Published:** 2012-07-13

**Authors:** Florence A Aeschlimann, Helene Werner, Oskar G Jenni, Rotraud K Saurenmann

**Affiliations:** 1 Children's Hospital, University of Zurich, Zurich, Switzerland

## Purpose

The so called growing pains (GP) are affecting 4-37% of all children with a peak incidence in the preschool years. The underlying cause of this medically harmless but perturbing condition is still unknown. Although parasomnias (e.g., sleep terrors, sleep talking, sleep walking) share several common features with GP such as age at onset, daytime of appearance, self-limited course and complete absence of symptoms on the following day, an association has not been established between the 2 conditions.

## Objective

To analyse the pain characteristics of children with GP and compare the sleep characteristics of the children with and without GP in order to investigate the possibility that GP constitute a parasomnia.

## Methods

The parents of 58 children with a diagnosis of GP according to the Peterson criteria filled a questionnaire about the characteristics of the GP and the sleep characteristics of their children. The study group was then further subdivided in 2 groups according to the time of pain onset: “evening GP” occurring already in the late afternoon and/or at bedtime, and “night GP” occurring only after falling asleep during the first half of the night. 38 children from a study about children’s sleep patterns served as the control cohort.

## Results

Children with GP had more difficulties waking up in the morning (p<0.0001), had more difficulties with re-entering sleep after waking up (p<0.0001), had a lower overall sleep quality (p=0.0002), used more commonly a transitional object (cuddly toy) (p= 0.002) and suffered more often from sleep terrors (p=0.005). In a multivariate analysis the factors wake-up difficulties, difficulties with re-entering sleep after waking up, sleep terrors and transitional object remained independently associated with GP. Then we compared the different variants of GP: 14 children (24%) qualified for the definition of “night GP” and 16 (28%) had “evening GP”. “Night GP” was significantly more common in boys (p=0.009), had fewer pain attacks during one night (p=0.04), were less likely to have their pain attacks following hectic days (p=0.04), had a better overall sleep quality (p=0.049) and also had more commonly sleep terrors (p= 0.1) than children with “evening GP”. In the multivariate analysis the factors gender, sleep terrors and occurrence after hectic days remained independently significant.

## Conclusion

Children with the so called GP have a disturbed sleep pattern. The highly significant association of growing pains, especially of the “night GP” variant, with sleep terrors supports the hypothesis of an association between these conditions and warrants further investigations.

## Disclosure

Florence A. Aeschlimann: None; Helene Werner: None; Oskar G. Jenni: None; Rotraud K. Saurenmann: None.

